# A comprehensive overview of exosome lncRNAs: Emerging biomarkers and potential therapeutics in gynecological cancers

**DOI:** 10.3389/fonc.2023.1138142

**Published:** 2023-03-17

**Authors:** Min Wang, Lulu Fu, Ying Xu, Shuai Ma, Xueying Zhang, Lianwen Zheng

**Affiliations:** Reproductive Medical Center, Department of Obstetrics and Gynecology, The Second Hospital of Jilin University, Changchun, China

**Keywords:** exosome lncRNA, expression, biomarkers, therapeutics, gynecological cancers

## Abstract

Ovarian, endometrial, and cervical cancer are common gynecologic malignancies, and their incidence is increasing year after year, with a younger patient population at risk. An exosome is a tiny “teacup-like” blister that can be secreted by most cells, is highly concentrated and easily enriched in body fluids, and contains a large number of lncRNAs carrying some biological and genetic information that can be stable for a long time and is not affected by ribonuclease catalytic activity. As a cell communication tool, exosome lncRNA has the advantages of high efficiency and high targeting. Changes in serum exosome lncRNA expression in cancer patients can accurately reflect the malignant biological behavior of cancer cells. Exosome lncRNA has been shown in studies to have broad application prospects in cancer diagnosis, monitoring cancer recurrence or progression, cancer treatment, and prognosis. The purpose of this paper is to provide a reference for clinical research on the pathogenesis, diagnosis, and treatment of gynecologic malignant tumors by reviewing the role of exosome lncRNA in gynecologic cancers and related molecular mechanisms.

## 1 Introduction

An exosome is a cell-secreted nanoscale vesicle containing DNA, proteins, lipids, RNA, metabolites, cytokines, transcription factor receptors, and other biologically active substances (1). Its composition is similar to that of parental cells and can be used as a “fingerprint” to identify relevant cells and provide specific signals that can be traced in circulating blood (2). The composition is similar to that of parental cells and can be used to identify relevant cells by providing specific signals that can be traced in circulating blood. Long noncoding RNAs (lncRNAs) are noncoding RNAs that are abundant in the cytoplasm and nucleus (3). They do not have protein-coding functions, but they can influence cancer development in a variety of ways and can be specifically sorted into the exosome (4). Despite the presence of RNA enzymes in the blood, lncRNAs can persist due to exosome protection (5, 6). Tumor-derived exosomes (TDE) lncRNAs can contribute to cancer progression in a variety of ways by altering the tumor microenvironment, the epithelial-mesenchymal transition (EMT), and angiogenesis, as well as playing a role in cancer growth maintenance and stabilization. Because cancer invasion, metastasis, treatment, and drug resistance are all intertwined, it is of great scientific importance to mine and explores the exosome lncRNAs that affect malignant biological behavior, as this can help to further investigate the mechanism of cancer development and provide new ideas and strategies for cancer treatment (7, 8).

## 2 Overview of the exosome

### 2.1 Discovery and distribution of exosome

**Figure 1:**
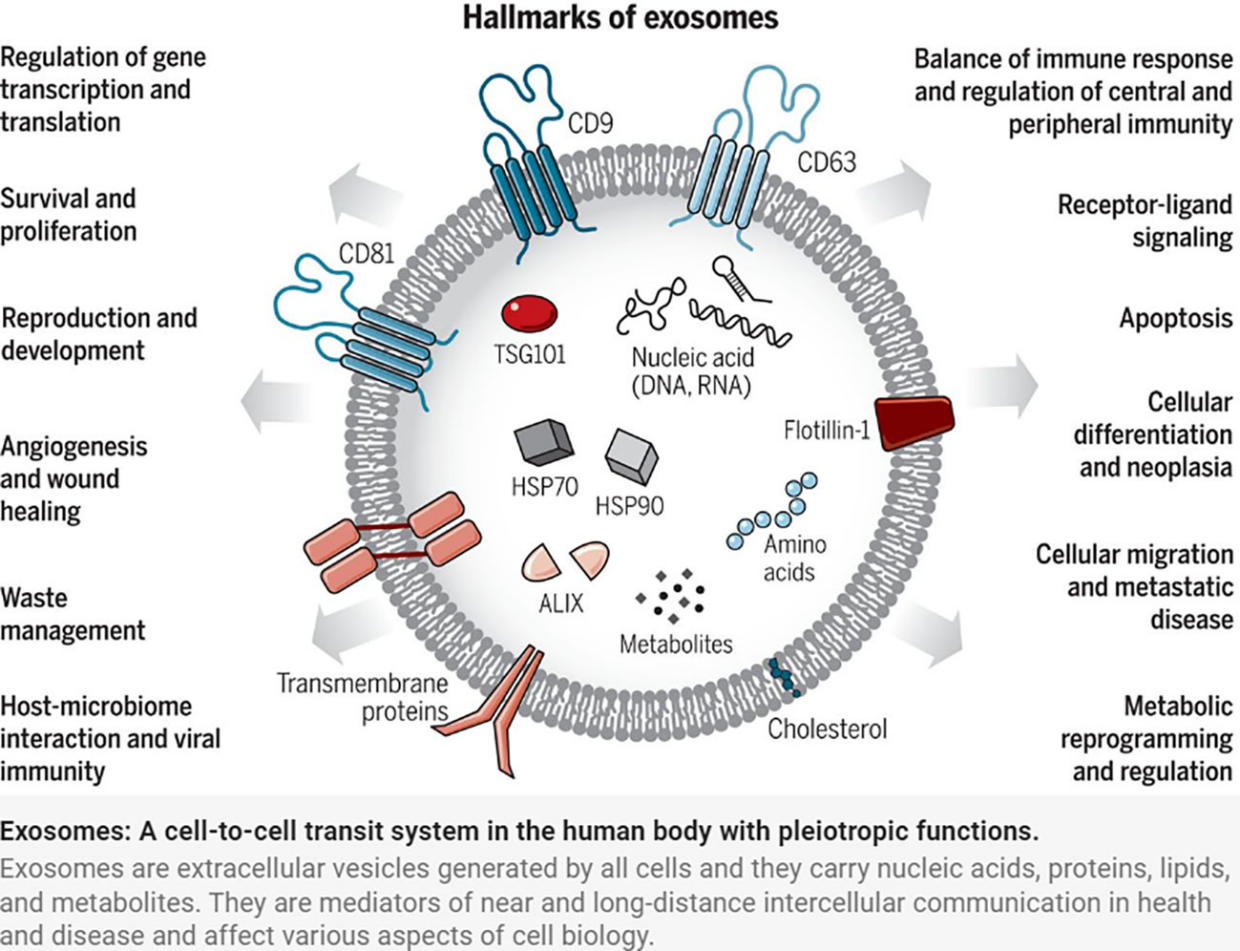
Exosomes: A cell-to-cell transit system in the human body with pleiotropic functions. Exosomes are extracellular vesicles generated by all cells and they carry nucleic acids, proteins, lipids, and metabolites. They are mediators of near and long-distance intercellular communication in health and disease and affect various aspects of cell biology (1).

Exosomes were discovered by Johnstone et al. in the study of extracellular cytoplasmic fusion of reticulocyte multivesicular bodies (9), are 30-150 nm in diameter (10), have a phospholipid bilayer structure, and belong to the extracellular vesicle family. Exosomes released from cells into the extracellular compartment are found in a variety of body fluids, including saliva, breast milk, blood, urine, amniotic fluid, and vaginal/alveolar lavage fluid (11). The endosomal sorting complex required for transport (ESCRT) is made up of the complexes ESCRT-0, I, II, and III, as well as co-proteins like apoptosis-linked gene 2-interacting protein X (ALIX) and vacuolar protein sorting 4 (VPS4). Several studies have confirmed the importance of ESCRT in exosome biosynthesis (12). Exosome production, on the other hand, is not entirely dependent on ESCRT mechanisms such as the ceramide mechanism. It was discovered that mouse oligodendrocytes secreted lipoprotein-carrying exosomes normally even after ESCRT inhibition and that cellular exosome secretion was reduced after ceramide synthesis inhibition, implying a regulatory role for ceramide in exosome synthesis. Exosomes are produced by cellular self-selection, and exosomes from different cells can carry different “cargo” (13). Under various physiological and pathological conditions, the same cell can produce multiple exosomes containing additional genetic information (14) (**Figure 1**).

### 2.2 Functions of exosome

Exosomes can create a pre-metastatic microenvironment suitable for cancer cell growth, regulate the glucose and lipid metabolism of target cells, counteract the body’s immune defense, and promote and cooperate with cancer development by transferring lncRNA to recipient cells and mediating material transport and information exchange.

Exosome has been confirmed as a circulating biomarker for various breast, colorectal, and bladder cancers in numerous studies (15). On January 21, 2016, the first exosome-based cancer diagnostic product was launched in the United States (16). The exosome is a natural lipid vesicle that can be used as a gene therapy carrier and has significant development potential in the field of cancer therapy (17). Exosomes can cross the blood-brain barrier and transport drugs and genes (e.g., proteins, lipids, DNA, and RNA) into tissues, effectively preventing their degradation (18). The drugs could not penetrate the blood-brain barrier in the control group of zebrafish embryos treated with conventional drugs, but in the experimental group, in which the anti-cancer drugs adriamycin and paclitaxel were integrated into the exosome and then introduced into zebrafish embryos, large number of exosomes could penetrate the blood-brain barrier and allow the drugs to reach the cancer cells directly (19). In advanced cancers, clinical trials targeting dendritic cell-derived exosomes (DEX) have been conducted (20). Cancer exosomes are known to play an essential role in the distant compartment effect, a recently discovered mechanism that effectively targets cancers and inhibits distant metastasis (21); As a result, the exosome is expected to be a novel and efficient drug delivery system. Exosomes can be used for gene therapy by transfecting siRNA into the exosome and successfully silencing genes using the exosome as a vector, according to research (22).

## 3 Overview of lncRNAs

### 3.1 Biogenesis of lncRNAs

**Figure 2:**
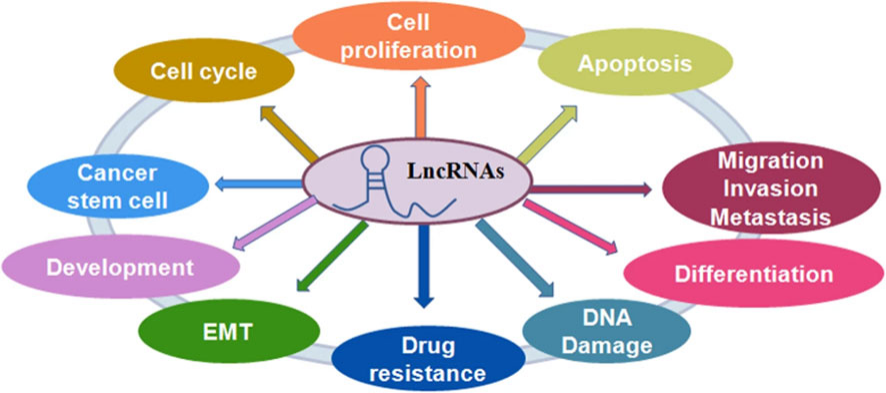
The role of lncRNAs in regulating cellular processes. LncRNAs play a critical role in the regulation of cell proliferation, cell apoptotic death, cell cycle, cell migration and invasion, epithelial-mesenchymal transition (EMT), cancer stem cells, DNA damage and drug resistance in cancer (25).

The noncoding region of the human genome contains approximately 88% of single nucleotide polymorphisms. Non-coding RNA is classified into two types based on its length: LncRNA and short-stranded noncoding RNA. LncRNA is a class of single-stranded RNA molecules with sizes less than 200 nt, the majority of which are found in the nucleus and some in the cytoplasm, and are classified as sense lncRNA, antisense lncRNA (AS lncRNA), bidirectional lncRNA, intronic lncRNA, and intergenic lncRNA (23). When compared to most protein-coding genes, lncRNAs have better cell specificity and relatively stable local secondary and tertiary structures, making them easier to detect in body fluids and capable of interacting with DNA, RNA, or proteins. They play an essential role in the physiological and pathological processes of the body (24). LncRNAs participate in a variety of biological pathways, including cell growth, by regulating gene transcription and post-translational expression. By regulating innate and adaptive immunity, lncRNAs can participate in a variety of immune pathways, and dysregulation of their expression levels can disrupt immune homeostasis. It is anticipated that it will be one of the most promising biomarkers for disease diagnosis and prognosis (25) (**Figure 2**).

### 3.2 LncRNAs are involved in gene expression

LncRNAs are important regulators at the epigenetic, transcriptional, and post-transcriptional levels (26). Epigenetic silencing or activation of target genes: lncRNAs can regulate gene expression at the epigenetic level *via* DNA methylation, demethylation, histone modification, and chromosome remodeling (27). MEG3 expression was found to be significantly reduced in glioblastoma due to DNA methyltransferase I-mediated hypermethylation of the MEG3 promoter, which downregulated MEG3 expression in glioblastoma and inhibited p53 protein activation.

Transcriptional level: Long noncoding RNAs (lncRNAs) can interact with transcription factors, enhancers, and promoters to regulate RNA transcription, localization, and stability (28). The lncRNA Gas5 can compete with the glucocorticoid response element (GRE) for binding to the glucocorticoid receptor (GR), preventing GR transcriptional activation and resulting in an autoimmune response. In breast cancer, low Gas5 expression increased cancer cells’ survival during starvation. P21-associated noncoding RNA with DNA damage activation (lncRNA PANDA) was found to promote osteosarcoma cell proliferation. Further research revealed that the lncRNA PANDA inhibited apoptosis in normal human fibroblasts by binding to transcription factors that prevented it from binding to apoptosis-related gene promoters. Long noncoding RNA homeobox (HOX) A11 antisense lncRNA (HOXA11-AS) was discovered to bind to transcription factor WD repeat domain 5 (WDR5) in the promoter region, promote -catenin transcription, and activate the Wingless-Type MMTV Integration Site Family (WNT) signaling pathway, accelerating cancer metastasis *in vivo*.

Post-transcriptionally, lncRNAs can form RNA dimers with target mRNAs *via* complementary base pairing, obstruct transcription factor binding, or directly recruit specific translation repressor proteins to regulate mRNA shearing, translation, and degradation (29). KLF4 is a transcriptional activator of vascular endothelial growth factor (VEGF). The lncRNA H19 can bind to miR-7, allowing miR-competitive endogenous RNA (ceRNA-7) to release translational repression of KLF4 and activate the KLF4/VEGF signaling pathway. Stable knockdown of exosome lncRNA H19 can significantly affect KLF4 and VEGF mRNA and protein expression levels, which affect the formation of the pre-metastatic microenvironment, inhibit cancer cell migration and invasion, and regulate the tumor microenvironment and vascular normalization. During the progression of hepatocellular carcinoma, the expression level of long noncoding RNA-activated by transforming growth factor beta (lncRNA-ATB) was increased and directly linked to IL-11, which altered IL-11 tertiary structure, increased the stability of IL-11 mRNA, induced IL-11 autocrine, triggered the signal transducer and activator of transcription 3 (STAT3) pathway, and promoted cancer metastasis and organ colonization. The first lncRNA with trans-activation, HOX transcript antisense RNA (HOTAIR), acts as a pro-oncogene in a variety of cancer cells, including breast cancer and hepatocellular carcinoma (30). HOTAIR, a lncRNA with sponge adsorption for miR-122, can regulate cancer cell epithelial-mesenchymal transition (31).

## 4 Exosome lncRNAs and tumor

**Figure 3:**
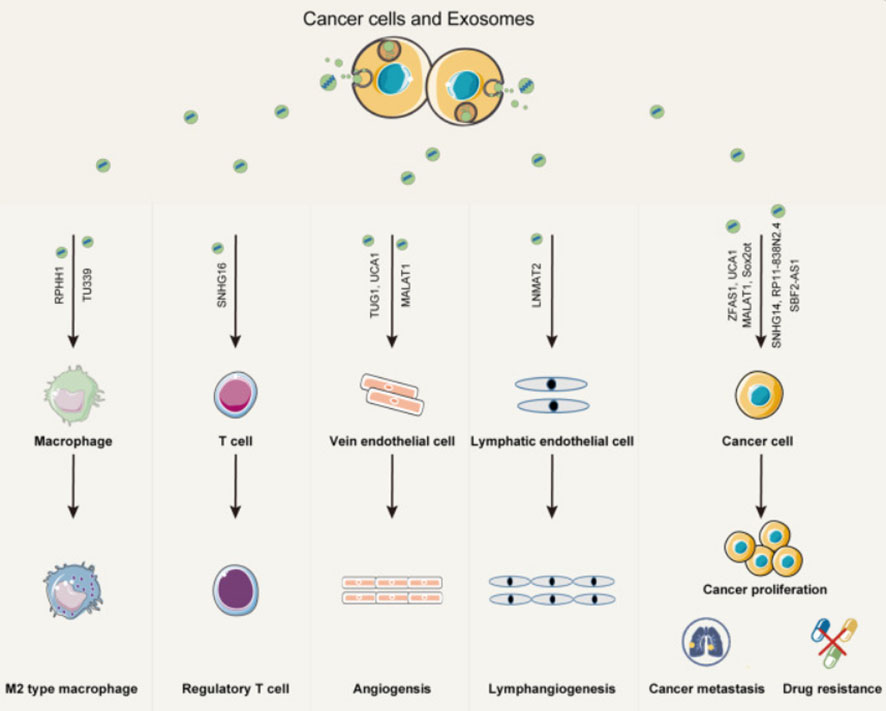
Exosomes play an important role in mediating the interaction between cancer cells and both immune cells and stromal cells within the cancer microenvironment. Exosomal lncRNAs from cancer cells can promote immune modulation, angiogenesis, cancer proliferation, metastasis, and drug resistance (35).

The early and precise diagnosis of malignant cancers has become a hot research topic. Cancer occurrence and progression are dependent on the interaction between cancer cells and the tumor microenvironment. In addition to intercellular contact and the release of soluble factors, cancer cells can communicate with the tumor microenvironment *via* exosomes (32). The TDE transports molecules such as content DNA, miRNA, and lncRNA that reflect genetic or signaling changes originating in cancer cells (33). lncRNA enters the recipient cells *via* the exosome and acts as a signaling mediator to coordinate cellular functions among cancer cells, creating a microenvironment conducive to cancer cell metastasis at a distant site (34–36). In cancer progression, lncRNAs can serve two purposes. MALAT1 (metastasis-associated lung adenocarcinoma transcript1) can promote or inhibit breast cancer metastasis by activating or inactivating neighboring prometastatic transcription factors (37). DEAD-box RNA helicase 3 (DDX3) also plays a dual role in the progression of lung cancer. On the one hand, DDX3 can activate the WNT signaling pathway, facilitating lung cancer metastasis. DDX3, on the other hand, can inhibit lung cancer progression by activating the MDM2/Slug/E-cadherin signaling pathway (38). The lncRNA HOTAIR can affect the co-localization and activity of vesicle-associated membrane protein 3 (VAMP3) and synaptosomal-associated protein 23 (SNAP23) to promote the fusion of MVB with the plasma membrane to promote HCC exosome secretion, confirming that lncRNAs have the function of promoting cancer exosome secretion and providing a new idea for the study of cancer lncRNAs (**Figure 3**).

### 4.1 Exosome lncRNAs and tumor microenvironment

The tumor microenvironment is made up of a variety of cells, including cancer cells and stromal cells like endothelial cells, fibroblasts, adipocytes, and mesenchymal stem cells (39). Tumorigenesis, progression, and metastasis are all affected by the characteristics of cancer cells as well as the interaction between cancer cells and stromal cells in the tumor microenvironment (40). The exosome, which is released into the extracellular environment *via* paracrine or autocrine signaling pathways and causes receptor cell-related phenotypic changes (27), is a critical communication mediator for primary tumor microenvironment alterations. Different exosome lncRNA sources play different roles (41). Cancer cells and tumor-associated macrophages (TAMs) may be important sources of exosomes in the tumor microenvironment (42). TDE, by remodeling the extracellular matrix (ECM) and inducing angiogenesis, creates a microenvironment favorable for cancer cell metastasis at distant sites (43). Cancer parenchymal cells use the exosome to transport biogenetic information to the extracellular space, transforming normal stromal cells and promoting cancer cell proliferation, apoptosis, migration, invasion, and prognosis (44, 45). Carcinoma-associated fibroblasts (CAFs), macrophages, and other cells secrete lncRNA-containing exosomes to promote cancer development and malignancy (46). LINC00092 was found to be significantly elevated in paraneoplastic fibroblasts in OC, along with elevated chemokine (C-X-C motif) ligand 14 (CXCL14), which was associated with metastasis and poor prognosis in OC (47).

### 4.2 Exosome lncRNA and tumor angiogenesis

The formation of neovascularization is an important environment for cancer genesis and development, and blood vessels provide sufficient oxygen and nutrients for cancer cell metastasis and growth (48). TDE can help with cancer angiogenesis and extracellular matrix remodeling by dynamically regulating different cells in the tumor microenvironment. The cancer vasculature is typically disorganized as a result of adjacent cancer cells compressing new blood vessels, resulting in tortuous and malformed vessels. Endothelial cells are loosely connected, and permeability and leakiness increase, allowing cancer cells to spread quickly into the vasculature and then develop distant metastases (49). As a result, anti-cancer cell angiogenesis will emerge as a novel therapeutic strategy. Cancer cells’ exosome lncRNA can act on endothelial cells in the microenvironment to promote cancer angiogenesis. Exosomes secreted by cancer stem cells invade endothelial cells, deliver lncRNA H19 to their target cells, and stimulate HUVEC angiogenesis by synthesizing and releasing VEGF (50). In preparation for cancer growth and metastasis, glioma cells were found to promote angiogenesis by increasing the expression of endothelial cell pro-angiogenic factor VEGFA *via* exosome lncRNA CCAT and lncRNA HOTAIR (51). The exosome lncRNA Small nucleolar RNA host gene 16 (SNHG16)/miR-4500/N-acetylgalactosamine-transferase 1 (GALNT1) axis has been linked to tumor angiogenesis.

### 4.3 Exosome lncRNAs and tumor metastasis

Metastasis is a fundamental challenge in cancer therapy because cancer cells and the tumor microenvironment regulate cancer proliferation and metastasis (52). TDE lncRNAs can promote malignant growth by interacting with the microenvironment and cancer cells, resulting in increased cancer proliferation and metastasis (53). During rapid growth, cancer cells cause internal tissue hypoxia and promote upregulation of hypoxia-inducible factor (HIF-1) expression, stimulating cancer cells to secrete exosomes with enhanced angiogenic and metastatic potential and promoting cancer invasion and metastasis (54). According to studies, exosome lncRNA 91H is highly expressed in patients’ serums with colorectal cancer and usually decreases after surgery. lncRNA 91H has been shown to promote cancer migration and invasion by regulating the expression of heterogeneous nuclear ribonucleoprotein K (HNRNPK) (55). HOTAIR, an exosome lncRNA, has been linked to bladder cancer progression, and knocking it out in uroepithelial bladder cancer cell lines inhibits EMT (56). MALAT1, an exosome-derived lncRNA that promotes cancer cell migration and prevents cancer cell apoptosis, was found to be positively related to the TNM stage and lymph node metastasis in NSCLC (57). It has been demonstrated that exosome-derived epidermal growth factor receptor (EGFR) protein in lung cancer cells induces the formation of tolerogenic dendritic cells (DCs), which in turn inhibits the anti-cancer effects of CD8+ T cells by inducing the production of regulatory T cells (Treg), and ultimately Treg promotes cancer immune escape (58). Elucidating the molecular mechanisms of cancer metastasis may lead to the development of more effective cancer therapeutic strategies (59).

### 4.4 Exosome lncRNA and cancer drug resistance

It is critical to investigate the specific mechanisms of innate or acquired drug resistance in cancer cells (60); Cancer cells and stromal cells in the tumor microenvironment can help spread cancer drug resistance by secreting exosomes (61). Exosomes can affect cell sensitivity to drugs *via* the following mechanisms (62). Exosomes directly wrap anti-cancer drugs, reducing their effectiveness. Exosomes transport bioactive molecules that compete for binding targets with anti-cancer drugs. Drug-resistant cells transmit drug-resistance information to sensitive cells *via* exosome-derived bioactive small molecules. Drug sensitivity information is transmitted from sensitive cells to drug-resistant cells *via* exosome-derived bioactive small molecules. Resistance to chemotherapeutic drugs could be improved by interfering with receptor cells with lncRNA, which could be a new therapeutic approach (63). lncRNA regulators of reprogramming (ROR) were found to be highly expressed in hepatocellular carcinoma cells (64). Drug resistance was found to be increased when hepatocellular carcinoma cells were treated with exosomes containing a high concentration of lncRNA ROR (65). Infection of lncRNA ROR in hepatocellular carcinoma cells with RNAi resulted in adriamycin sensitivity, and cancer cells may use exosomes and lncRNA to enhance drug resistance in nearby cells. Celastrol is thought to be therapeutic for a variety of cancers. When compared to free celastrol and celastrol exosome preparations, anti-cancer activity was significantly increased, with no liver or nephrotoxicity (66). Paclitaxel-resistant breast cancer cells can be induced by delivering the lncRNA SNHG15 to sensitive cells *via* exosomes. The exosome lncRNA KCNQ1OT1 is a critical molecule mediating radiotherapy resistance in lung cancer A549 cells. The use of CAFs as an entry point for reversing radiotherapy resistance in lung cancer cells provides a critical theoretical foundation. Investigating the effect of exosome lncRNAs on drug resistance will aid in elucidating the molecular mechanism of cancer drug resistance and provide new ideas for overcoming or reversing drug resistance (67).

## 5 Exosome lncRNA and gynecologic malignancies

### 5.1 Exosome lncRNA and ovarian cancer

Ovarian cancer (OC) is the most difficult to diagnose and has the worst prognosis of all malignant cancers of the female reproductive system, causing serious health problems in women (68). The pathogenesis of OC is complex, the early clinical symptoms are subtle, and the metastatic potential is high. When most patients are diagnosed, they are already in an advanced stage of the disease (69), so radical surgery cannot be used, the treatment effect is inadequate, and more than 70% of OC patients have a recurrence. The Food and Drug Administration (FDA) has approved only two biomarkers, CA125 and HE4, as diagnostic biomarkers for OC (70). CA125 is widely used in clinical settings, but it has some limitations (71). CA125, for example, is less sensitive in early-stage OC and can be elevated in pregnancy, pelvic inflammatory disease, endometriosis, and other conditions. In the presence of conditions such as acute and chronic renal insufficiency, HE4 can also indicate gynecological diseases and abnormal changes (72). As a result, a new reliable marker is required for the early detection of OC (73).

Exosome lncRNA can be used as a non-invasive diagnostic and screening tool, requiring only a small amount of fresh or frozen blood from OC patients and simultaneously analyzing for DNA, RNA, and protein. Exosomes can be extracted from the urine and blood of OC patients using a human recombinant S100A8 protein aptamer bound to cell membrane HSP70 (74). CD24 was found in exosomes from malignant ascites and *in vitro* cancer cells. This marker has been used to predict the prognosis of OC, demonstrating the exosome’s utility as a minimally invasive biopsy (75). Experiments with magnetic nanobeads revealed that many HER2-positive exosomes were found in the serum of OC patients (76). Chen et al. (77) discovered that CA125 levels were higher in exosomes than in serum, that serum exosome-derived CA125 improved the sensitivity of OC diagnosis, and that serum HE4 combined with exosome CA125 improved the diagnostic efficiency of OC. Zhang et al. (78) examined the circulating exosomes in the plasma of OC patients and identified seven biomarkers with diagnostic ability, including HER2, EGFR, Folate Receptor (FR), CA-125, Epithelial Cell Adhesion Molecule (EpCAM), CD24, and (CD9+CD63), and demonstrated that these exosome biomarkers not only distinguished OC patients from benign subjects but also differentiated early and advanced OC, indicating the MALAT1 is a long noncoding RNA that is involved in the angiogenesis and metastasis of OC. Sun et al. (79) discovered that the lncRNA MALAT1 plays an important role in the development of OC by mediating the Janus kinase 2 (JAK2)/STAT3 signaling pathway, promoting OC cell proliferation, and inhibiting cancer cell apoptosis. Jin et al. (80) discovered that the lncRNA MALAT1 could increase OC cell proliferation while inhibiting cancer cell apoptosis *via* the PI3K-protein kinase B (PKB, AKT) signaling pathway, enhancing OC cell invasion, migration, and EMT function. Some researchers discovered that the expression level of serum exosomes (81) was higher when testing the expression of serum exosome MALAT1. MALAT1 expression was significantly higher in epithelial OC patients than in controls, and it was associated with an advanced International Federation of Gynecology and Obstetrics (FIGO) stage, a high histological grade, and lymph node metastasis. Increased serum exosomeMALAT1 expression was associated with a progressive metastatic epithelial OC phenotype and a poor prognosis, suggesting that it could be used as a prognostic or predictive biomarker for epithelial OC. The HOXA transcript at the distal tip (HOTTIP), a homeobox lncRNA, is critical in the progression of OC. HOTTIP overexpression was found to increase IL-6 expression and secretion in OC cells. IL-6 activated the STAT3 pathway by binding to IL-6 receptors on the surface of neutrophils surrounding cancer cells, increasing the expression of PD-L1 on the surface of neutrophils, inhibiting T cell activity further, accelerating OC immune escape, and ultimately promoting cancer cell growth and metastasis (82). The lncRNA NEAT1 was found to be significantly overexpressed in ovarian cancer cells compared to normal human ovarian epithelial cells. Through sponge adsorption of miR-36, lncRNA NEAT1 may promote ovarian cancer cell proliferation by upregulating fibroblast growth factor (FGF) 9.

The use of exosomes for vaccine preparation is a novel approach in cancer immunotherapy. TDE has low immunogenicity, a low drug attrition rate, and easy tissue diffusion, making it suitable for use as a drug or gene carrier for targeting OC and as a cancer vaccine to inhibit cancer growth. The cytotoxicity of paclitaxel-loaded macrophage exosomes against drug-resistant P-gp transfected Manin-Darby canine kidney epithelial cells (MDCKMDR1) cell line was increased more than 50-fold, and the anti-cancer effect of the drug-loaded exosomes was demonstrated (83). Farrukh et al. (84) discovered that exosome delivery of anthocyanin had a strong therapeutic effect on both drug-sensitive and drug-resistant human ovarian cancer cells and that its therapeutic activity was synergistically enhanced when combined with cisplatin. The co-culture of the hypoxic OC cell line exosome (HEX) with cancer cells during cisplatin treatment improved cell survival, according to Kalpana et al. (85). Simultaneously, a known inhibitor, STAT3, inhibited exosome release. Exosome release and cisplatin treatment increased apoptosis, indicating that HEX can promote OC metastasis and increase chemoresistance, and could be a new mechanism for cancer metastasis and chemoresistance, as well as a therapeutic intervention to improve clinical outcomes.

### 5.2 Exosome lncRNA and endometrial carcinoma

Endometrial carcinoma (EC) is one of the most common malignant cancers of the female reproductive system (86), accounting for 20%–30% of all malignancies of the female genital tract. In recent years, the incidence of EC has been increasing year after year, and the age of onset has gotten younger. EC can be diagnosed clinically based on symptoms such as vaginal bleeding or increased fluid discharge, but a definitive diagnosis requires further examination improvement. Fear of diagnostic scraping and hysteroscopy causes some patients to postpone their investigation, delaying the best time for diagnosis and treatment. Those who do not receive timely treatment at an early stage frequently have poor prognoses and survival rates. When the presence of lesions in the endometrium is determined through diagnostic scraping, the distribution of lesions cannot be accurately grasped, and small local lesions may be missed, increasing the rate of EC misdiagnosis. The clinical treatment of EC is primarily surgical, with the decision to combine radiotherapy based on high-risk factors. There are few adjuvant treatment options for advanced and recurrent cancers. As a result, identifying practical early diagnostic markers and precise therapeutic targets is critical.

Exosome lncRNA regulates EC proliferation and invasion primarily through angiogenesis, EMT, and immune regulation, among other things. Exosome lncRNA promotes the formation of a tumor microenvironment by transforming related cells, which not only speeds up normal cell proliferation but also changes the biological characteristics of nearby and distant non-cancer cells, allowing cancer cells to spread. Through related signaling pathways, some lncRNAs can effectively promote EC cell proliferation and enhance EC cell invasion, migration, and EMT function, thereby promoting cancer growth (87). Some lncRNAs, on the other hand, can effectively inhibit cancer cell proliferation, block the cell cycle process, and promote cancer cell apoptosis *via* related signaling pathways, which may be related to the composition of the tumor microenvironment, particularly CAFs (88).

MEG3 is a long noncoding RNA with anti-cancer properties (89). Reduced expression of MEG3, which inhibits cancer cell proliferation, migration, and invasion and promotes apoptosis, has been linked to cancer development and progression (90, 91). MEG3 activity is controlled by both TP53-dependent and TP53-independent mechanisms. The TP53 gene is mutated in the majority of human cancers, and it functions as a transcription factor, controlling the expression of many target genes and thus inhibiting cancer development and growth. The differential expression of TP53 in normal and cancerous tissues suggests that MEG3 could be used to assess cancer staging and prognosis (92). Guo et al. examined the expression of MEG3 and Notch signaling molecules in EC tissues and cell lines using real-time quantitative PCR and Western blotting. MEG3 expression was found to be significantly downregulated in EC tissues, whereas Notch protein expression was found to be upregulated in both. MEG3 downregulation inhibits EC proliferation by inhibiting the Notch signaling pathway (93). In EC patients, low expression of exosome lncRNA MEG3 in plasma predicts more high-risk factors, a higher recurrence rate, and a worse prognosis. By comparing the serum expression levels of lncRNA ROR and miR-29 in EC patients and healthy women and using ROC curves to assess the diagnostic value of both in EC, Serum lncRNA ROR and miR-29 levels were found to be significantly higher in EC patients than in healthy women. The expression levels in patients with TNM stages I–II increased dramatically, indicating the combined serum level (94). MALAT1 overexpression is associated with a poor prognosis for EC, implying that MALAT1 could be used as a novel biomarker and diagnostic target for EC (95).

The primary issue with cancer drug therapy is cancer drug resistance, particularly in recurrent cancers where acquired drug resistance renders the therapeutic effect ineffective. Exosome-mediated lncRNA communication in the tumor microenvironment has been shown in studies to be one of the reasons for increased drug resistance. It is possible to inhibit the production or uptake of an exosome-carrying “oncogene” and promote the production or uptake of an exosome-carrying “oncogene” based on the fact that exosomes can transport proteins and nucleic acids related to cancer invasion, metastasis, angiogenesis, and drug resistance. This opens up a new avenue for the future use of exosomes in the treatment of EC. The engineered exosome is more effective in targeting therapy than the original exosome, and it also reduces cytotoxicity and significantly inhibits tumor growth (96). Exosome lncRNAs play an important role in the development of EC, opening up new avenues for the early diagnosis and treatment of EC patients. They may also become an important tool in monitoring the progression and prognosis of EC in the future.

### 5.3 Exosome lncRNA and cervical cancer

Cervical cancer (CC) is one of the most common cancers in women. According to data, more than 500,000 people are diagnosed with CC each year, with the majority of deaths occurring in developing countries (97, 98). As a result, CC is a global public health issue that should not be underestimated (99). Despite the fact that chemotherapy combined with targeted therapy can improve overall survival (OS), progression-free survival (PFS), and cancer mortality in CC patients, cancer incidence continues to rise. The prognosis of advanced CC, which has a low local control rate and is prone to distant metastasis, is influenced by high-risk factors. The treatment effect is frequently poor, with only a 60% 5-year survival rate (100). As a result, improving early diagnosis of CC, identifying therapeutic targets, and investigating biomarkers that can indicate prognosis have emerged as top priorities in CC basic and clinical research (101).

Because of the impact of exosome LncRNA on the tumor microenvironment and its biological properties, it has the potential to become a cancer biomarker for CC patients, which has important clinical implications in cancer screening, treatment detection, and prognosis evaluation (102). Exosomes from HeLa cells in CC have been shown to promote distant metastasis by inducing endothelial cell endoplasmic reticulum stress and disrupting vascular endothelial cell integrity, thereby disrupting tight endothelial junctions (103). When CC HeLa cell exosomes were injected into mice, they found increased vascular permeability and cancer metastasis. The primary mechanism involved the CC HeLa cell exosome regulating the expression of closed junction proteins. Immunity against CC was improved in a mouse model of CC by increasing the cytotoxic activity of DEX-induced CD8+ T cells against cancer cells, prompting CD8+ T cell proliferation, and increasing IFN secretion (104).

HOXA11 is a recently discovered and researched lncRNA (105). HOXA11-AS has been shown in studies to promote cancer cell proliferation by regulating the expression of miR-124, miR-140-5p, LATS1, PADI2, and other genes (106–108). Exosome lncRNA HOXA11-AS may increase the expression of SRY-related high-mobility group box 4 (SOX4) in endothelial cells, increasing the proliferative capacity of endothelial cells involved in cervical cancer. According to the ROC curve, the specificity of lncRNA gradually increased during hepatocarcinogenesis (GIHCG), and the sensitivity was 88.75% in distinguishing between healthy people and CC patients. In the future, lncRNA GIHCG could be used to predict CC (109). HOTAIR and MALAT1 lncRNAs were found to be significantly overexpressed in exosomes isolated from the lavage fluid of CC patients (111.112). The lncRNA MEG3 was found to be significantly reduced and correlated with cancer stage, metastasis, and other factors. Chen et al. demonstrated that MEG3 can inhibit cervical cancer cell proliferation, invasion, and migration by regulating the Rac1 and PI3K/AKT/MMP-2/9 signaling pathways (110). When compared to non-neoplastic cervical tissues, the expression of lncRNA MEG3 was significantly downregulated in the histopathological grading of cervical intraepithelial neoplasia (CIN) in CIN 2 and CIN3. According to one study, MEG3 expression was reduced in cervical tissues, and it was associated with cancer size, lymph node metastasis, high-risk HPV infection, and the FIGO stage. *In vitro*, ectopic expression of MEG3 may inhibit the proliferation of human CC cells HeLa and C-33A. The researchers discovered that NF-kappaB interacting lncRNA (NKILA) inhibits proliferation and promotes apoptosis in cervical squamous cells by down-regulating miRNA-21 expression. LncRNA ArfGAP with the RhoGAP domain, ankyrin repeat, and PH domain 1 antisense RNA (ARAP1-AS1) can promote proto-oncogene c-Myc translation in cervical cancer by separating dimers and promoting tumorigenesis (59). Furthermore, through interactions with recombinant Polypyrimidine Tract Binding Protein 1 (PTBP1), LncRNA surfactant associated 1 (SFTA1P) promoted the degradation of tropomyosin 4 (TPM4) mRNA and the progression of cervical cancer (52). These findings support MEG3’s critical role in the molecular etiology of CC and point to MEG3’s potential use in the treatment of CC (111) (**Table 1**).

**Table 1:** The expression and functions of exosomal lncRNAs in gynecological cancers.

Cancer types	Specimen source	Exosomal lncRNAs	Functions	References
OC	Cells	LINC00092	Metastasis	(47)
OC	Cells	MALAT1	Proliferation	(79)
OC	Cells	HOTTIP	Metastasis	(82)
OC	Cells	MEG3	Drug resistance	(36)
OC	Cells	GIHCG	Proliferation	(34)
OC	Cells	PTAR	Metastasis	(34)
OC	Cells	MORT	proliferation	(73)
OC	Cells	HOTAIR	Metastasis	(86)
OC	Cells	NEAT1	proliferation	(86)
OC	Cells	H19	Proliferation	(86)
OC	Cells	HOXA11	Biomarkers	(105)
EC	Cells	MEG3	Proliferation	(93)
EC	Cells	ROR	Proliferation	(94)
EC	Cells	MALAT1	Biomarkers	(95)
EC	Cells	DLEU1	Metastasis	(87)
EC	Cells	HOTAIR	Metastasis	(86)
EC	Cells	NEAT1	Metastasis	(86)
EC	Cells	H19	Proliferation	(86)
CC	Cells	ARAP1-AS1	Proliferation	(59)
CC	Cells	NKILA	Proliferation	(59)
CC	Cells	MORT	proliferation	(73)
CC	Cells	HOXA11	Proliferation	(105)
CC	Cells	GIHCG	Biomarkers	(109)
CC	Vaginal Douche	HOTAIR	Metastasis	(112)
CC	Vaginal Douche	MALAT1	Metastasis	(113)
CC	Vaginal Douche	MEG3	Metastasis	(110)
CC	Cells	LINC01305	Metastasis	(102)
CC	Cells	NEAT1	Proliferation	(86)
CC	Serum	H19	Biomarkers	(101)
CC	Cells	SPRY4-IT1	Proliferation	(111)
CC	Cells	GAS5	Proliferation	(111)
CC	Cells	PVT1	Proliferation	(111)
CC	Cells	LINC00675	Metastasis	(111)
CC	Cells	CCST1	Proliferation	(111)
CC	Cells	ZEB1-AS1	Metastasis	(111)

## 6 Conclusion

Exosomal lncRNA has a wide range of research applications (114). Exosome lncRNAs regulate a variety of pathophysiological processes, including cancer cell genesis, invasion, metastasis, and vascular neogenesis, as well as mediate cancer drug resistance and play an important role in cancer development. Exosome lncRNA is thought to be a novel marker for gynecologic cancer diagnosis, efficacy evaluation, and prognosis prediction. Exosome lncRNA research is still in its early stages, and its functions are not fully understood. There are the following flaws: The specificity of exosome lncRNA as a molecular marker for gynecological tumor diagnosis has yet to be determined; The technology of exosome *in vitro* synthesis as a carrier of targeted therapeutic drugs has yet to be improved; Exosome lncRNAs play a role in a variety of cancers, and multiple exosome lncRNAs have the same cancer action target, but their interaction is still unknown, making it difficult to fully resolve their regulatory network. The above problems can be solved one by one with the rapid development of proteomics, high-throughput sequencing, transcriptomics, and bioinformatics analysis, and researchers will have a better understanding of the mechanisms of exosome-derived lncRNAs in the development of gynecological malignancies and their clinical applications.

## Author contributions

MW, LF, YX, SM, XZ, and LZ performed literature searches and selected the studies and reviews discussed in the manuscript. The first draft of the manuscript was prepared by MW, LF, YX, SM, and XZ and made subsequent amendments. LZ revised the manuscript. All authors contributed to the article and approved the submitted version.
